# Proteomics of Deep Cervical Lymph Nodes After Experimental Traumatic Brain Injury

**DOI:** 10.1089/neur.2023.0008

**Published:** 2023-05-26

**Authors:** Noora Puhakka, Shalini Das Gupta, Sara Leskinen, Mette Heiskanen, Janika Nättinen, Ulla Aapola, Hannu Uusitalo, Asla Pitkänen

**Affiliations:** ^1^ A. I. Virtanen Institute for Molecular Sciences, University of Eastern Finland, Kuopio, Finland.; ^2^ Eye and Vision Research, Faculty of Medicine and Health Technology, Tampere University, Tampere, Finland.; ^3^ Tays Eye Centre, Tampere University Hospital, Tampere, Finland.

**Keywords:** animal studies, proteomics, traumatic brain injury

## Abstract

Traumatic brain injury (TBI) damages the glymphatic-lymphatic system. We hypothesized that brain injury associated with trauma results in the enrichment of brain-relevant proteins in deep cervical lymph nodes (DCLNs), the end station of meningeal lymphatic vessels, and that some of these proteins will present mechanistic tissue biomarkers for TBI. Proteomes of rat DCLNs were investigated in the left DCLN (ipsilateral to injury) and right DCLN at 6.5 months after severe TBI induced by lateral fluid percussion injury or after sham operation. DCLN proteomes were identified using sequential window acquisition of all theoretical mass spectra. Group comparisons, together with functional protein annotation analyses, were used to identify regulated protein candidates for further validation and pathway analyses. Validation of a selected candidate was assessed using enzyme-linked immunosorbent assay. Analysis comparing post-TBI animals with sham-operated controls revealed 25 upregulated and 16 downregulated proteins in the ipsilateral DCLN and 20 upregulated and 28 downregulated proteins in the contralateral DCLN of post-TBI animals. Protein class and function analyses highlighted the dysregulation of enzymes and binding proteins. Pathway analysis indicated an increase in autophagy. Biomarker analysis suggested that a subgroup of post-TBI animals had an increase in zonula occludens-1 coexpressed with proteins linked to molecular transport and amyloid precursor protein. We propose here that, after TBI, a subgroup of animals exhibit dysregulation of the TBI-relevant protein interactome in DCLNs, and that DCLNs might thus serve as an interesting biomarker source in future studies aiming to elucidate pathological brain functioning.

## Introduction

Traumatic brain injury (TBI) occurs in all sexes and age groups, and approximately 70 million persons annually are estimated to suffer these injuries worldwide.^1^ Primary injury is the immediate physical manifestation of the brain trauma.^2^ Secondary injury occurs hours to days after the impact-related primary injury and affects several cerebral and peripheral cellular, chemical, and molecular processes. Previous studies revealed that TBI disrupts the signaling of gap junctions, ion channels, neurotransmitters and their receptors, mitochondrial function, blood–brain barrier (BBB) function, calcium homeostasis, and immune responses.^3^ Clinically useful pharmacotherapies that improve recovery from TBI are not currently available (reviewed previously^4^). The discovery of biomarkers describing the condition of the brain during secondary injury is an unmet clinical need that would greatly contribute to the development of potential treatments for TBI.

Metabolic waste and immune cells from the central nervous system are proposed to flow through a pathway consisting of the glymphatic system and meningeal lymphatics into the deep cervical lymph nodes (DCLNs).^5–7^ Soon after conceptualization of the glymphatic system, Plog and colleagues^8^ studied how certain putative TBI biomarkers are transported from the cerebrospinal fluid to the blood. They demonstrated, in a murine TBI model, that these markers are transported through the glymphatic system and cervical lymphatics.^8^ In the present study, we used sequential window acquisition of all theoretical mass spectra (SWATH-MS) to identify the post-TBI proteome of rat DCLNs. We hypothesized that the DCLN proteome represents the dysregulation of protein networks and pathways linked to post-TBI sequelae in the brain (e.g., chronic amyloid pathology) and can serve as a source for mechanistic biomarkers.

## Methods

### Animals and model of traumatic brain injury

Details of the study design and animal numbers are presented in Supplementary Figure S1A Severe TBI was induced by lateral fluid percussion injury (FPI) in 30 adult male Sprague-Dawley rats as previously described.^9,10^ From the full animal cohort, 12 TBI rats, 8 sham-operated rats, and 8 naïve rats were included in the present study. The animal procedures were approved by the Animal Ethics Committee of the Provincial Government of Southern Finland. All animal procedures were performed by competent personnel according to European Union legislation concerning the use of animals for scientific purposes (Directive 2010/63/EU).

### Dissection of deep cervical lymph nodes

At 6.5 months post-TBI, rats were deeply anesthetized by intraperitoneal injection of an anesthetic cocktail (6 mL/kg), containing sodium pentobarbital (58 mg/kg), magnesium sulfate (127.2 mg/kg), propylene glycol (42.8%), and absolute ethanol (11.6%) and perfused with 0.9% saline solution (30 mL/min for 5 min), to remove blood from the tissues. Within 20 min of the perfusion, DCLNs from both sides of the neck were dissected and cleared from surrounding tissue under a microscope (Leica CLS 150 XE; Leica Microsystems, Wetzlar, Germany), as reviewed previously^11^ (Supplementary Fig. S1B). Thereafter, DCLNs were flash-frozen in liquid nitrogen and stored at −70°C.

### Sequential window acquisition of all theoretical mass spectra proteomics

For proteomics analysis, DCLNs were homogenized and lysed, and the total protein concentration was measured. From each sample, 50 μg of protein was subjected to reduction, alkylation, and tryptic digestion.^12^ Thereafter, 4 μg of each individual sample was injected into mass spectrometry using SWATH-MS. A peptide ion library representing 1850 rat DCLN proteins was generated using 36 samples originating from all study groups. Relative quantification of the proteins was performed with the Triple-TOF 5600+ mass spectrometer (Sciex, Framingham, MA), equipped with Eksigent 425 Nano-LC using SWATH acquisition. Each sample was measured twice.

### Enzyme-linked immunosorbent assay for zonula occludens-1

Enzyme-linked immunosorbent assays (ELISAs) were performed according to the manufacturer's instructions using a commercial kit for the detection of rat zonula occludens-1 (ZO-1; CSB-E17287r; Cusabio, Houston, TX). For the analysis, lymph node homogenate supernatants were diluted 1:10 in the sample dilution buffer.

### Statistical analyses

Bioinformatics analyses and data visualization are summarized in the supplementary methods (Supplementary File S1). To identify the differentially expressed proteins, independent samples *t*-test and mean fold change (FC) values were calculated in R software (R Foundation for Statistical Computing, Vienna, Austria). FC cutoffs of >1.5 and <0.67 were applied after this step. All other statistical analyses were conducted in GraphPad Prism software (version 9; GraphPad Software Inc., La Jolla, CA). The Mann-Whitney U test was used to assess differences in ELISA results. Receiver operating characteristic (ROC) analysis was performed to estimate the biomarker potential of ZO-1. The multiple unpaired *t*-test was used to assess differentially expressed proteins in TBI subgroups after ZO-1 ELISA.

## Results

### Rat deep cervical lymph nodes contained cerebral cortex-enriched proteins

The SWATH-MS analysis successfully quantified 1512 proteins in rat DCLNs (Supplementary File S2 The majority of these proteins were metabolite- (*n* = 305) or protein-modifying (*n* = 171) enzymes (Supplementary Fig. S2A). The most common molecular functions of detected proteins were binding (*n* = 552) and catalytic activity (*n* = 501; Supplementary Fig. 2B). Most of the detected proteins exhibited no tissue specificity. Interestingly, however, the tissue with the highest number of enriched proteins present in our samples was the cerebral cortex (*n* = 27; Supplementary Fig. 2C). Further, the database used in TissueEnrich software indicated that the gene expression levels of many cerebral cortex-enriched proteins were relatively low in lymph node tissue (Supplementary Fig. 2D).

To summarize, the DCLN proteome is rich in enzymes and proteins with binding capacity. We also detected brain-enriched proteins in DCLNs, indicating their possible clearance through the glymphatic system.

### Increased autophagy after traumatic brain injury in rat deep cervical lymph nodes

**FIG. 1.**
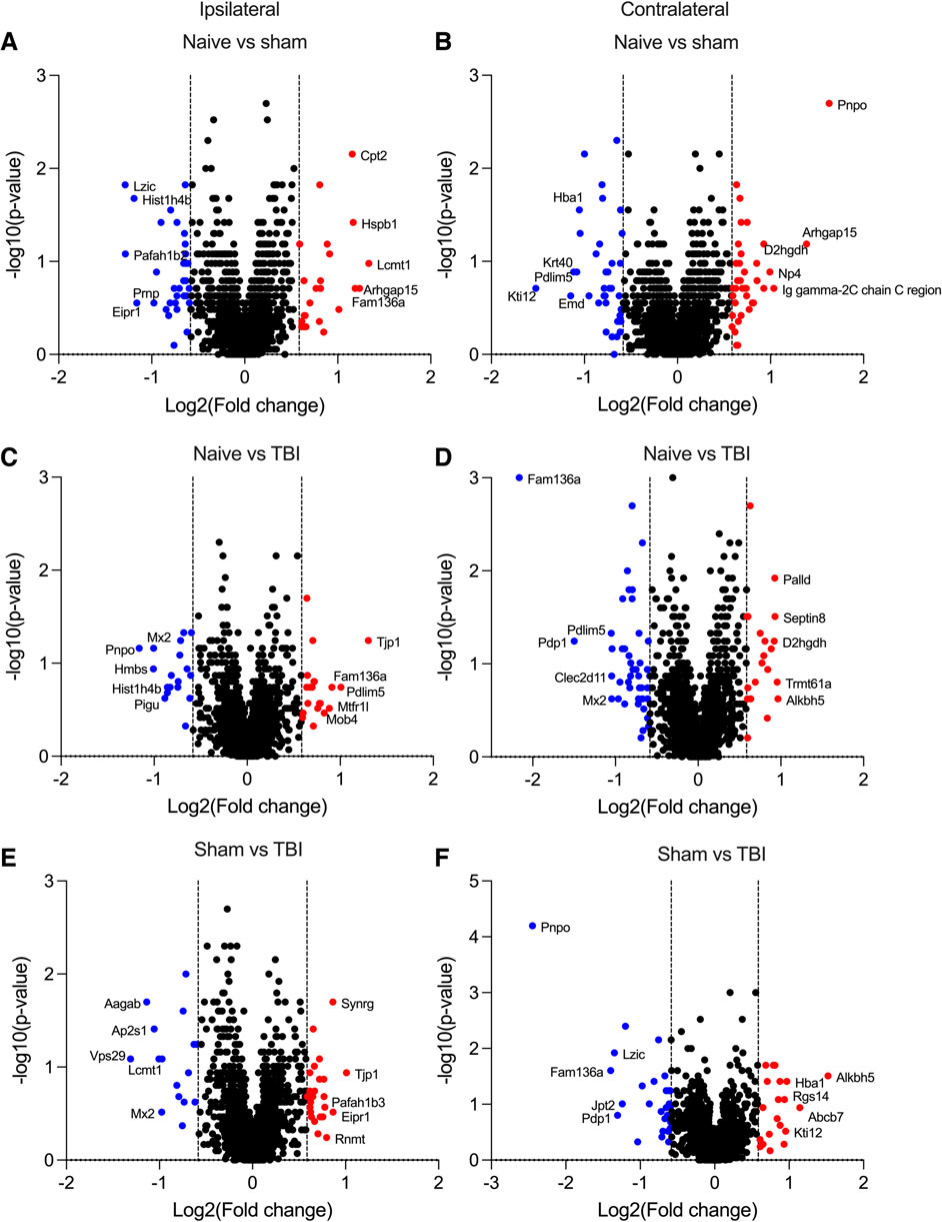
Dysregulated proteins in rat deep cervical lymph nodes (DCLNs) after traumatic brain injury (TBI). Upregulated (FC >1.5, dashed line on the right) and downregulated (FC <0.667, dashed line on the left) proteins in DCLNs (**A**) ipsilaterally and (**B**) contralaterally in sham-operated rats compared with naïve animals. Upregulated (FC >1.5, dashed line on the right) and downregulated (FC <0.667, dashed line on the left) proteins in DCLNs (**C**) ipsilaterally and (**D**) contralaterally in post-TBI rats compared with naïve animals. Upregulated (FC >1.5, dashed line on the right) and downregulated (FC <0.667, dashed line on the left) proteins in DCLNs (**E**) ipsilaterally and (**F**) contralaterally in post-TBI rats compared with sham-operated animals. FC, fold change.

Principal component analysis or unsupervised clustering revealed no major differences between study groups (Supplementary Fig. S3A–D). Differential expression analysis, however, revealed 24 upregulated and 32 downregulated proteins ipsilaterally and 38 upregulated and 37 downregulated proteins contralaterally in sham-operated animals compared with naïve rats (Fig. 1A,B; Supplementary File S2; Supplementary Fig. S3E). Moreover, differential expression analysis revealed 20 upregulated and 18 downregulated proteins ipsilaterally and 20 upregulated and 46 downregulated proteins contralaterally in TBI rats compared with naïve animals (Fig. 1C,D; Supplementary File S2; Supplementary Fig. S3E). Further, we observed 25 upregulated and 16 downregulated proteins ipsilaterally and 20 upregulated and 28 downregulated proteins contralaterally in TBI rats compared with sham-operated animals (Fig. 1E,F; Supplementary File S2; Supplementary Fig. S3E).

**FIG. 2.**
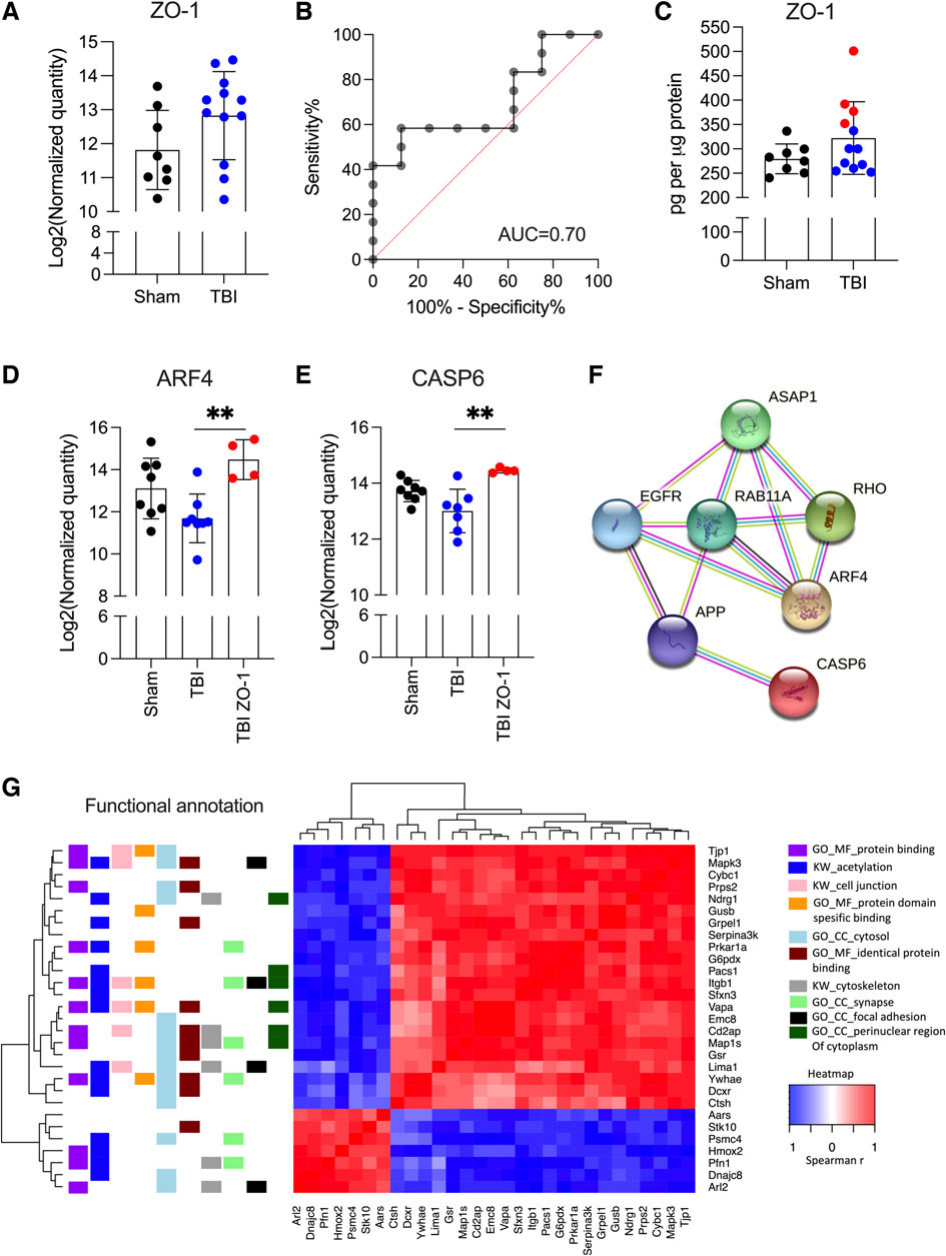
Dysregulation of the zonula occludens-1 (ZO-1) interactome in rat deep cervical lymph nodes (DCLNs) in a subgroup of animals after traumatic brain injury (TBI). (**A**) Relative quantitation of ZO-1 with sequential window acquisition of all theoretical mass spectra ipsilaterally in sham-operated rats and post-TBI animals. (**B,C**) Analysis of protein quantitation using ELISA and a receiver operating characteristic test (AUC = 0.70) of ZO-1 suggested increased levels of the protein in a subgroup of rats after TBI (red dots). (**D,E**) Statistical analysis highlighted the upregulation of ARF4 (*p* < 0.01) and CASP6 (*p* < 0.01) when these 4 rats were compared with the remaining TBI animals (*n* = 8). (**F**) Protein-protein network (String; https://string-db.org/) analysis revealed ASAP1, RHO, EGFR, RAB11A, and APP to be the five most closely connected proteins for ARF4 and CASP6. (**G**) Functional annotation of ZO-1-coexpressed proteins highlighted proteins related to protein binding, acetylation, cell junction, protein domain-specific binding, cytosol, identical protein binding, cytoskeleton, synapse, focal adhesion, and proteins located in the perinuclear region of the cytoplasm. APP, amyloid precursor protein; ARF4, ADP ribosylation factor 4; ASAP1, ArfGAP with SH3 domain, ankyrin repeat, and PH domain 1; CASP6, caspase 6; CC, cellular compartment; EGFR, epidermal growth factor receptor; ELISA, enzyme-linked immunosorbent assay; GO, gene ontology; KW, keyword; MF, molecular function; TBI, traumatic brain injury.

All group comparisons (Supplementary Figs. S4–S6) highlighted a dysregulation of enzymes and binding proteins. No major dysregulation of tissue-specific proteins was detected. Lymph-node–enriched proteins, however, were regulated after TBI, suggesting that brain trauma also alters peripheral lymphatic proteins (Supplementary Fig. S7). Pathway analysis of regulated proteins, together with treemap visualization, suggested increased autophagy (Supplementary Fig. S8).

In summary, although the overall proteome of DCLNs was not remarkably different at 6.5 months after experimental TBI, pathway analysis indicated a possible increase in proteins involved in autophagy.

### Dysregulated cell junction protein interactome in deep cervical lymph nodes is related to synapses, protein transport, and amyloid precursor protein after traumatic brain injury

One of the most highly dysregulated proteins after TBI was ZO-1 (also known as tight junction protein 1), which showed a 2.01-fold increase compared with sham-operated experimental controls (Fig. 2A). Mean concentration of ZO-1 in DCLN samples was 322 ± 74 pg/μg of protein (range, 252–501; median, 300) in the TBI group and 280 ± 31 pg/μg of protein (range, 240–336; median, 279) in the sham group (1.15-fold, *U*_8,12_ = 29, *p* = 0.1569) when assessed with ELISA. ROC analysis after ELISA quantified ZO-1 levels in the same samples indicated a moderate biomarker potential (area under the curve [AUC] = 0.70; 95% confidence interval = 0.46–0.93; *n* = 8 sham-operated, *n* = 12 TBI; Fig. 2B). Based on the ROC analysis, we selected a subgroup of TBI animals (*n* = 4; ELISA quantitation >340 pg/μg of protein; Fig. 2C) for further protein coexpression analysis.

First, rats exhibiting an increase in ZO-1 were compared with the other post-TBI rats. Statistical analysis highlighted the upregulation of ADP ribosylation factor 4 (ARF4; 1.24-fold, *t*_8,4_ = 5.153, *df* = 10, *p* = 0.001971; Fig. 2D) and caspase 6 (CASP6; 1.12-fold, *t*_8,4_ = 3.915, *df* = 10, *p* = 0.002889; Fig. 2E), two proteins that are directly related to RAB11A and amyloid precursor protein (APP; Fig. 2F). Next, we identified proteins that were coexpressed with ZO-1 in the correlation analysis. Cell junction proteins (6 of 26; adjusted *p* value [adj.*p*] = 0.0045), proteins binding to the protein domain (6 of 26; adj.*p* = 0.0055), and perinuclearly located cytosolic proteins (6 of 26; adj.*p* = 0.077) were functional annotation classes among ZO-1-coexpressing proteins. Other functional annotation classes were protein binding (12 of 26; adj.*p* = 0.00076), acetylation (15 of 26; adj.*p* = 0.0012), cytosol (15 of 26; adj.*p* = 0.0082), identical protein binding (10 of 26; adj.*p* = 0.022), cytoskeleton (6 of 26; adj.*p* = 0.076), synapse (6 of 26; adj.*p* = 0.077), and focal adhesion (4 of 26; adj.*p* = 0.077).

In summary, a subgroup of post-TBI rats seemed to have a dysregulated cell junction protein interactome in the DCLNs. This interactome is linked to APP, synapses, and cytosolic protein transport.

## Discussion

Waste from the central nervous system is proposed to flow through a pathway comprising the glymphatic system and meningeal lymphatics into the DCLNs.^5–7^ Here, we aimed to identify any brain-derived proteins accumulating in DCLNs after TBI that could be potentially used as tissue biomarkers for TBI-induced cellular pathology.

We detected brain-enriched proteins in the DCLNs of all study groups, indicating their possible clearance throughout the glymphatic system. Iliff and colleagues^13^ demonstrated that chronic impairment of glymphatic pathway function might be why the post-traumatic brain is vulnerable to tau aggregation and, thereafter, to further neurodegeneration. Previous studies^14^ indicated that in a mouse model of Alzheimer's disease (APP/PS1 mice), brain protein clearance is disrupted, even before the visible accumulation of beta-amyloid. To explore the function of dysregulated DCLN protein content beyond the differentially expressed protein lists, we performed pathway analyses and functional annotations. First, we investigated the immune response, a well-known contributor to TBI pathophysiology that can last for months to years in both the lateral FPI model and humans with TBI and has been linked to persisting neurological symptoms.^15–18^ Contrary to our expectations, immune response-related functions were not regulated in DCLNs at the chronic 6.5-months' post-injury sampling point. Despite this, autophagy was one of the highlighted cellular functions showing an increase. Importantly, autophagy flux is reported to be increased in the brain tissue of rat TBI models.^19,20^

As summarized by Zetterberg and Blennow,^21^ putative molecular biomarkers for TBI are typically searched from either the cerebrospinal fluid, blood, or other body fluids. We explored whether relatively easily accessible DCLNs could serve as a new source for biomarker harvesting. For example, DCLN proteins could be used to monitor waste clearance using protein-tagged imaging tracers or collection of cervical lymph fluid for protein analysis. Also, these molecules could serve as biomarkers for monitoring of efficacy therapeutic approaches aimed at improving meningeal lymphatic drainage in order to enhance post-TBI recovery. For this first piloting study, we selected one post-TBI time point (6.5 months) relevant for our hypothesis. Based on bioinformatics analysis, ZO-1 levels in DCLNs were quantified using ELISA. Interestingly, a subgroup of rats with TBI exhibited high ZO-1 levels. Levels of ZO-1 are closely associated with the degree of BBB damage and an indicator of BBB destruction (reviewed previously^22^). Detection of ZO-1 is also reported in the lymphatic endothelial junction of peripheral lymph nodes.^23^ Thus, it remains to be explored whether post-TBI elevation of ZO-1 reports on DCLN pathology, rather than on chronic BBB pathology, in the brain.

Functional annotation of ZO-1 coexpressed proteins revealed dysregulation of cytosolic protein transport and other cell junction proteins. Animals with high ZO-1 levels also had increased levels of ARF4 and CASP6, proteins that belong to the same protein network as APP. These protein networks have been previously connected to epilepsy and Alzheimer's disease, the well-known comorbidities of TBI.^24,25^ The association of the increased levels of ZO-1 in DCLNs with the post-TBI outcome, however, remains to be investigated in future pre-clinical and clinical studies.

### Conclusions and study limitations

Here we provide the first catalogue of rat DCLN proteins for experimental TBI. A subgroup of animals after TBI exhibited chronic dysregulation of the TBI-relevant protein interactome in DCLNs, including ZO-1, a marker of BBB dysfunction. The association of increased post-TBI levels of ZO-1 (or its network proteins) with brain pathology or behavioral outcome needs to be addressed in more comprehensive pre-clinical studies with a larger sample size, greater temporal granularity, and using different injury severities to pave the way for a clinical assessment of the translational potential of DCLN proteins in clinical applications.

## Supplementary Material

Supplemental data

Supplemental data

Supplemental data

Supplemental data

Supplemental data

Supplemental data

Supplemental data

Supplemental data

Supplemental data

Supplemental data

## Data Availability

Raw proteomics data are provided along the article in **Supplementary File S2**.

## Abbreviations Used

adj.*p*: adjusted *p* value
APP: amyloid precursor protein
ARF4: ADP ribosylation factor 4
AUC: area under the curve
BBB: blood–brain barrier
CASP6: caspase 6
DCLNs: deep cervical lymph nodes
ELISA: enzyme-linked immunosorbent assay
FC: fold change
FPI: fluid percussion injury
ROC: receiver operating characteristic
SWATH-MS: sequential window acquisition of all theoretical mass spectra
TBI: traumatic brain injury
ZO-1: zonula occludens-1
