# Supplementary material for: Proteomics of Deep Cervical Lymph Nodes After Experimental Traumatic Brain Injury
Source: Neurotrauma Rep. 2023 May 26;4(1):359–66. doi: 10.1089/neur.2023.0008 (PMC10240307; doi:10.1089/neur.2023.0008)

**Supplementary Figure 1. Study design.** **(A)** From the full animal cohort, 12 TBI rats, 8 sham-operated rats, and 8 naïve rats were included in this study. At the time of the injury, the mean body weight of the rats was 341.3±15.6 g (range 305-378 g; median 340 g; 1 value missing). Mean hit pressure for TBI induction was 2.90±0.12 atm (range 2.54–3.12 atm; median 2.92 atm; 1 value missing). The detected mean post-impact apnea time was 25.7±10.6 s (range 10–60 s; median 25 s). For sham-operated animals, the righting reflex was 3.21±1.11 min post-anesthesia (range 1–5 min; median 3 min; 1 value missing). For TBI rats, the righting reflex was 16.26 ± 10.29 min post-anesthesia (range 7–60 min; median 14 min; 3 values missing). Acute mortality in this study was 10% (3/30). One rat was excluded from the study after TBI induction because the dura was broken. **(B)** Representative image describing the sampled deep cervical lymph nodes (dashed line on the left and right sides). Abbreviations: SWATH-MS, Sequential Window Acquisition of All Theoretical Mass Spectra; TBI, traumatic brain injury.


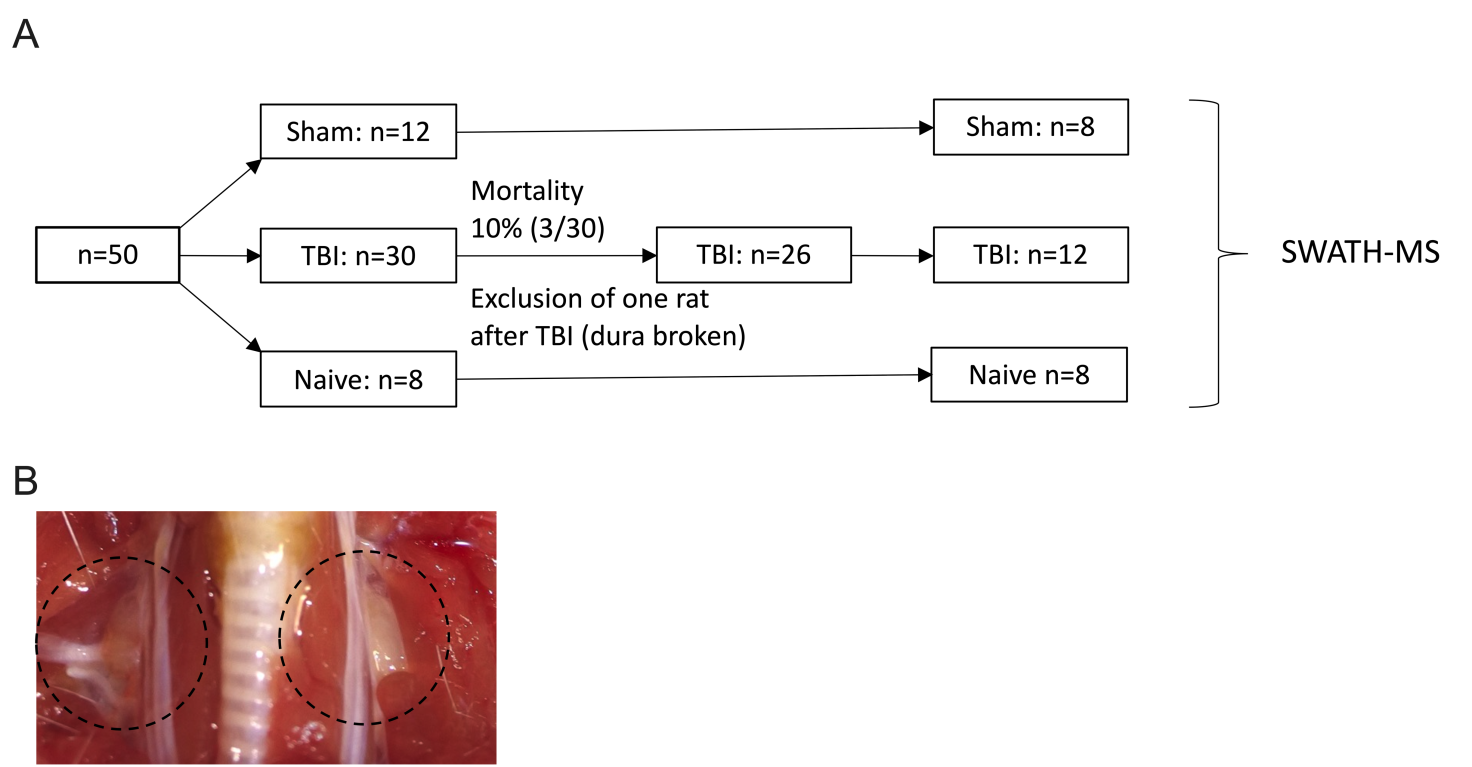

Supplement: Supplemental data [file Supp_FigS1.docx]
