# Supplementary material for: Proteomics of Deep Cervical Lymph Nodes After Experimental Traumatic Brain Injury
Source: Neurotrauma Rep. 2023 May 26;4(1):359–66. doi: 10.1089/neur.2023.0008 (PMC10240307; doi:10.1089/neur.2023.0008)
Supplement: Supplemental data [file Supp_FileS1.docx]

**Supplementary File1. Supplementary Methods**

**Proteomics of Deep Cervical Lymph Nodes after Experimental TBI**

Noora Puhakka^1,4*^, Shalini Das Gupta^1,5^, Sara Leskinen^1,6^, Mette Heiskanen^1,7^, Janika Nättinen^2,8^, Ulla Aapola^2,3,9^, Hannu Uusitalo^2,3,10^, Asla Pitkänen^1,11^

*^1^A. I. Virtanen Institute for Molecular Sciences, University of Eastern Finland, PO Box 1627, FI-70211 Kuopio, Finland*

*^2^Eye and Vision Research, Faculty of Medicine and Health Technology, Tampere University, FI-33014 Tampere University, Finland*

*^3^Tays Eye Centre, Tampere University Hospital, FI-33520 Tampere, Finland*

*^4^* noora.puhakka@uef.fi

*^5^* shalini.dasgupta91@gmail.com

*^6^* sarales@student.uef.fi

*^7^* mette.heiskanen@uef.fi

*^8^* janika.nattinen@tuni.fi

*^9^* ulla.aapola@tuni.fi

*^10^* hannu.uusitalo@tuni.fi

*^11^* asla.pitkanen@uef.fi

**Supplementary Methods**

**Bioinformatics and data visualization**

The Swiss-prot database was used to create ion libraries by ProteinPilot 4.7 software (Sciex, Redwood City, CA, USA). SWATH mapping on a library was performed with PeakView and MarkerView (Sciex). To determine the protein class and molecular function of the detected proteins, we used PANTHER (version 17.0; Rattus norvegicus; (1)). To assess the tissue specificity of the detected genes, we used TissueEnrich (2). For this, we converted rat protein symbols with Ensembl BioMart to human symbols. Principal component analyses were conducted with R package “stats”. Heatmaps were created with R package “pheatmap”, using Ward’s clustering method and Pearson’s correlation distance measure. Venn diagrams were drawn with Venny 2.1 (<https://bioinfogp.cnb.csic.es/tools/venny/index.html>). The treemap visualization was achieved with the R package “treemap”. Data for treemap was generated with QIAGEN IPA (core analysis, version 68752261, QIAGEN Inc., Aarhus, Denmark). A correlation heatmap was generated using R package “gplots”. Functional annotations included in the correlation heatmap panel were generated with DAVID (3).

**References**

1. Mi H, Muruganujan A, Casagrande JT, Thomas PD. Large-scale gene function analysis with the PANTHER classification system. Nat Protoc. 2013 Aug;8(8):1551–66.

2. Jain A, Tuteja G. TissueEnrich: Tissue-specific gene enrichment analysis. Bioinformatics [Internet]. 2019 Jun 1;35(11):1966–7. Available from: https://doi.org/10.1093/bioinformatics/bty890

3. Huang DW, Sherman BT, Lempicki RA. Systematic and integrative analysis of large gene lists using DAVID bioinformatics resources. Nat Protoc [Internet]. 2009 Jan 1 [cited 2018 May 31];4(1):44–57. Available from: http://www.ncbi.nlm.nih.gov/pubmed/19131956
