# Supplementary material for: Proteomics of Deep Cervical Lymph Nodes After Experimental Traumatic Brain Injury
Source: Neurotrauma Rep. 2023 May 26;4(1):359–66. doi: 10.1089/neur.2023.0008 (PMC10240307; doi:10.1089/neur.2023.0008)

**Supplementary File 1. Supplementary methods.**

**Supplementary File 2. Raw and analyzed proteomics data with sample numbers.**

**Supplementary Figure 2. Proteins detected in rat deep cervical lymph nodes (DCLNs).** **(A)** Sequential Window Acquisition of All Theoretical Mass Spectra (SWATH-MS) analysis quantified 1512 proteins belonging most commonly to protein classes of metabolite interconversion enzyme (n=305 proteins) and protein modifying enzyme (n=171 proteins). **(B)** Annotation of molecular functions revealed binding (n=552 proteins) and catalytic activity (n=501 proteins) to be most common among the detected proteins. **(C)** The tissue with had highest number of enriched proteins present among detected proteins was the cerebral cortex (n=27). **(D)** Tissue enrichment analysis indicated that gene expression (TPM value for RNA) of many of these proteins was relatively low in lymph node tissue.


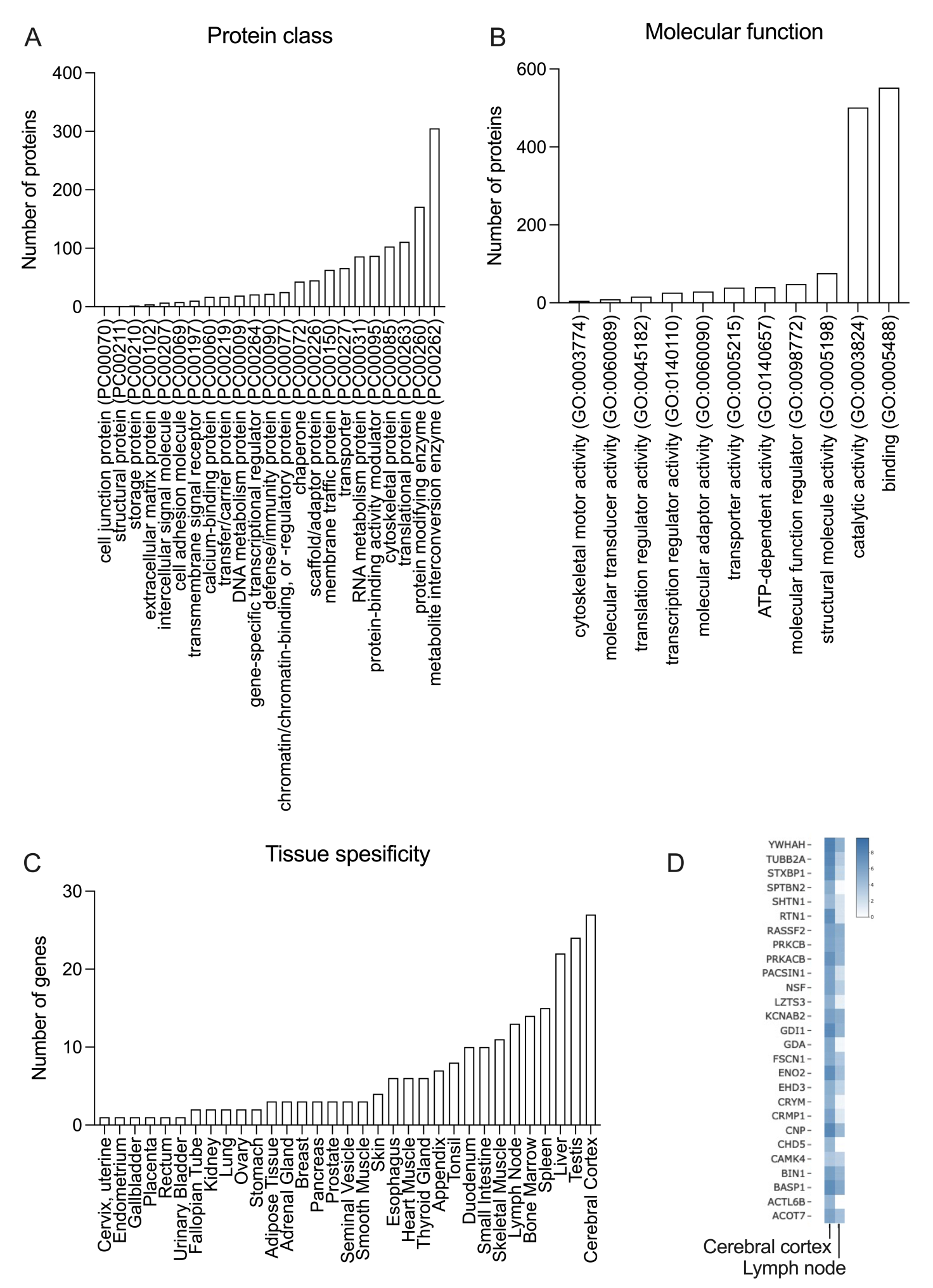

Supplement: Supplemental data [file Supp_FigS2.docx]
