# Supplementary material for: Proteomics of Deep Cervical Lymph Nodes After Experimental Traumatic Brain Injury
Source: Neurotrauma Rep. 2023 May 26;4(1):359–66. doi: 10.1089/neur.2023.0008 (PMC10240307; doi:10.1089/neur.2023.0008)

**Supplementary Figure 3. Differential expression analysis of proteins in rat deep cervical lymph nodes (DCLNs) after traumatic brain injury (TBI).** The principal component analysis did not reveal differences between the groups **(A)** ipsilaterally or **(B)** contralaterally. Similarly, we observed no major clustering of groups **(C)** ipsilaterally or **(D)** contralaterally when unsupervised hierarchical clustering was used to identify group separation. **(E)** Differential expression analysis showed 31 downregulated and 20 upregulated proteins ipsilaterally when sham-operated rats were compared to naïve animals. There were 18 downregulated and 25 upregulated proteins ipsilaterally when post-TBI rats were compared with naïve animals. From these, 8 downregulated and 3 upregulated proteins were the same between these 2 comparisons. Similarly, contralaterally, 37 and 46 proteins were decreased in sham-operated and post-TBI rats compared with naïve animals. On the contralateral side, the numbers of upregulated proteins were 37 and 19 when sham-operated and post-TBI rats were compared with naïve animals. From these, 12 downregulated and 9 upregulated proteins were the same between these 2 comparisons. Abbreviations: TBI, traumatic brain injury.


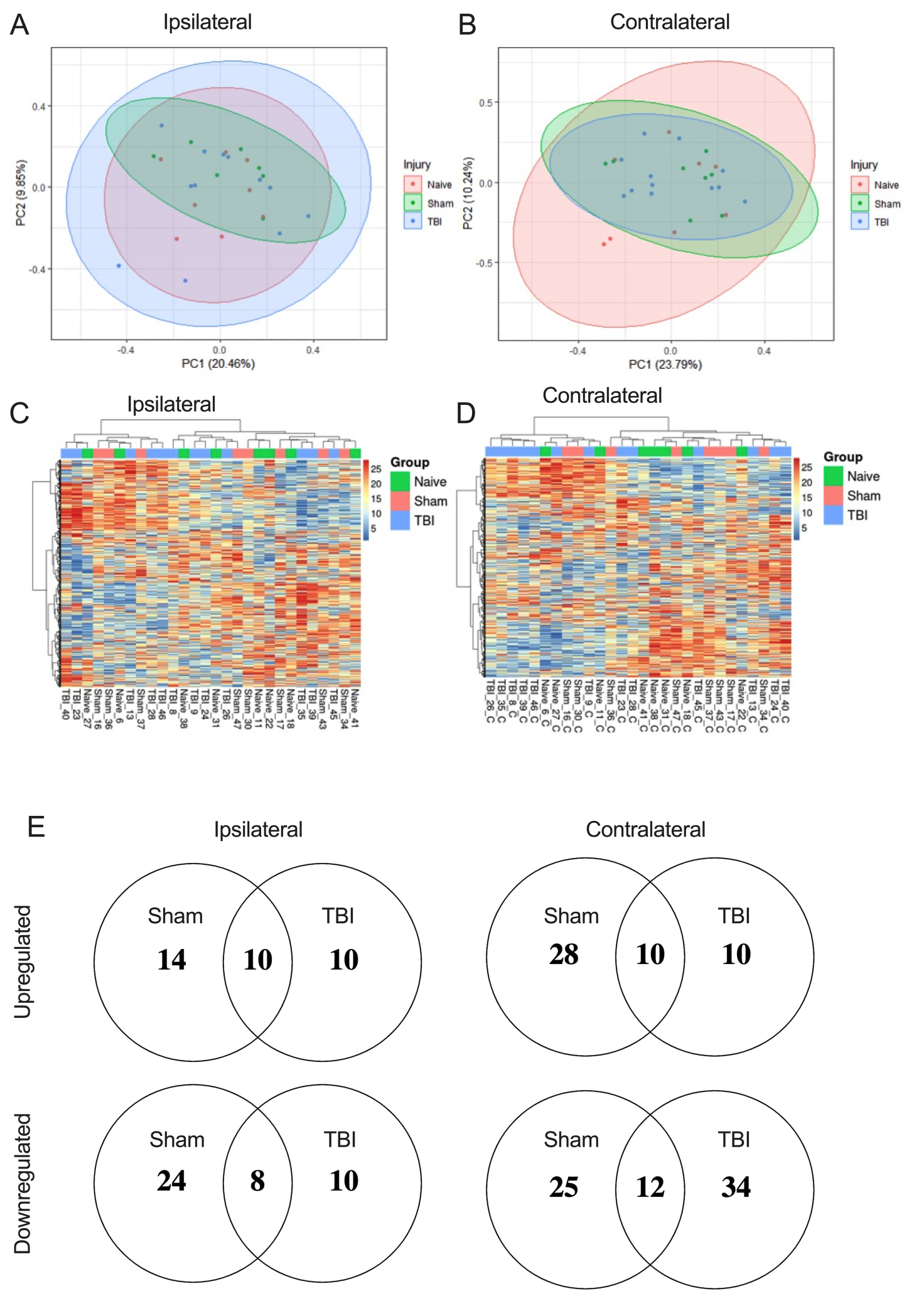

Supplement: Supplemental data [file Supp_FigS3.docx]
