# Supplementary material for: Proteomics of Deep Cervical Lymph Nodes After Experimental Traumatic Brain Injury
Source: Neurotrauma Rep. 2023 May 26;4(1):359–66. doi: 10.1089/neur.2023.0008 (PMC10240307; doi:10.1089/neur.2023.0008)
Supplement: Supplemental data [file Supp_FigS4.docx]

**Supplementary Figure 4. Most regulated protein classes and molecular functions of regulated proteins of sham-operated rats compared with naïve animals.**

**
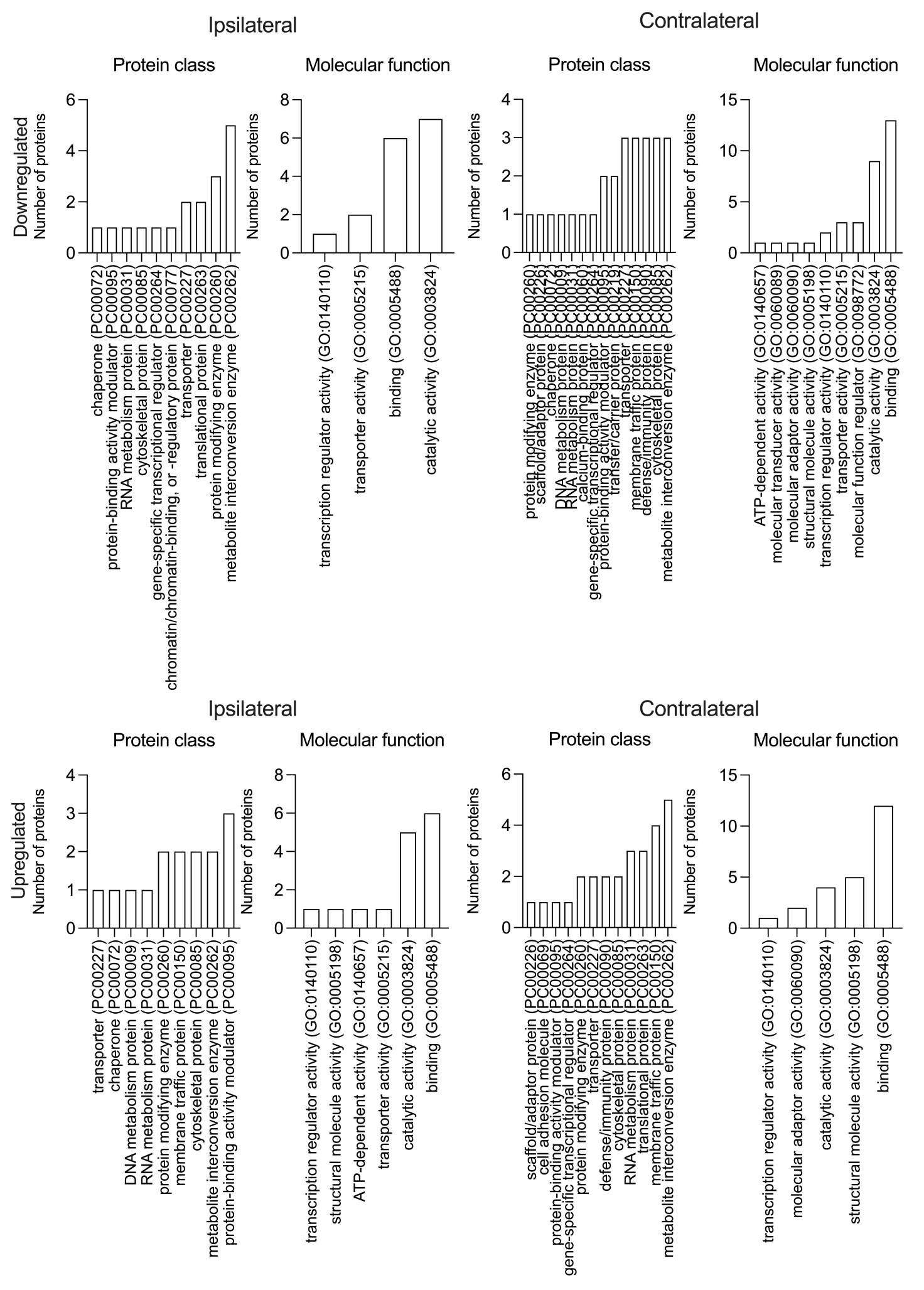
**
