# Supplementary material for: Proteomics of Deep Cervical Lymph Nodes After Experimental Traumatic Brain Injury
Source: Neurotrauma Rep. 2023 May 26;4(1):359–66. doi: 10.1089/neur.2023.0008 (PMC10240307; doi:10.1089/neur.2023.0008)

**Supplementary File 1. Supplementary methods.**

**Supplementary File 2. Raw and analyzed proteomics data with sample numbers.**

**Supplementary Figure 8. Treemap visualization of major pathways dysregulated in rat deep cervical lymph nodes (DCLNs) after traumatic brain injury (TBI).** Combined ipsilateral and contralateral sham compared with TBI biologic functions from Qiagen IPA presented as a treemap. The main rectangles (for example “cellular movement”) represent the main categories. The sub-rectangles represent the disease and function annotations belonging to a certain category (for example “chemotaxis” in “cellular movement”). Their sizes are proportional to the associated z-score: the bigger the rectangle, the larger the deviation from 0. Also, the color of the rectangles represents the z-score: blue indicates z<0 (downregulated) and red indicates z>0 (upregulated).


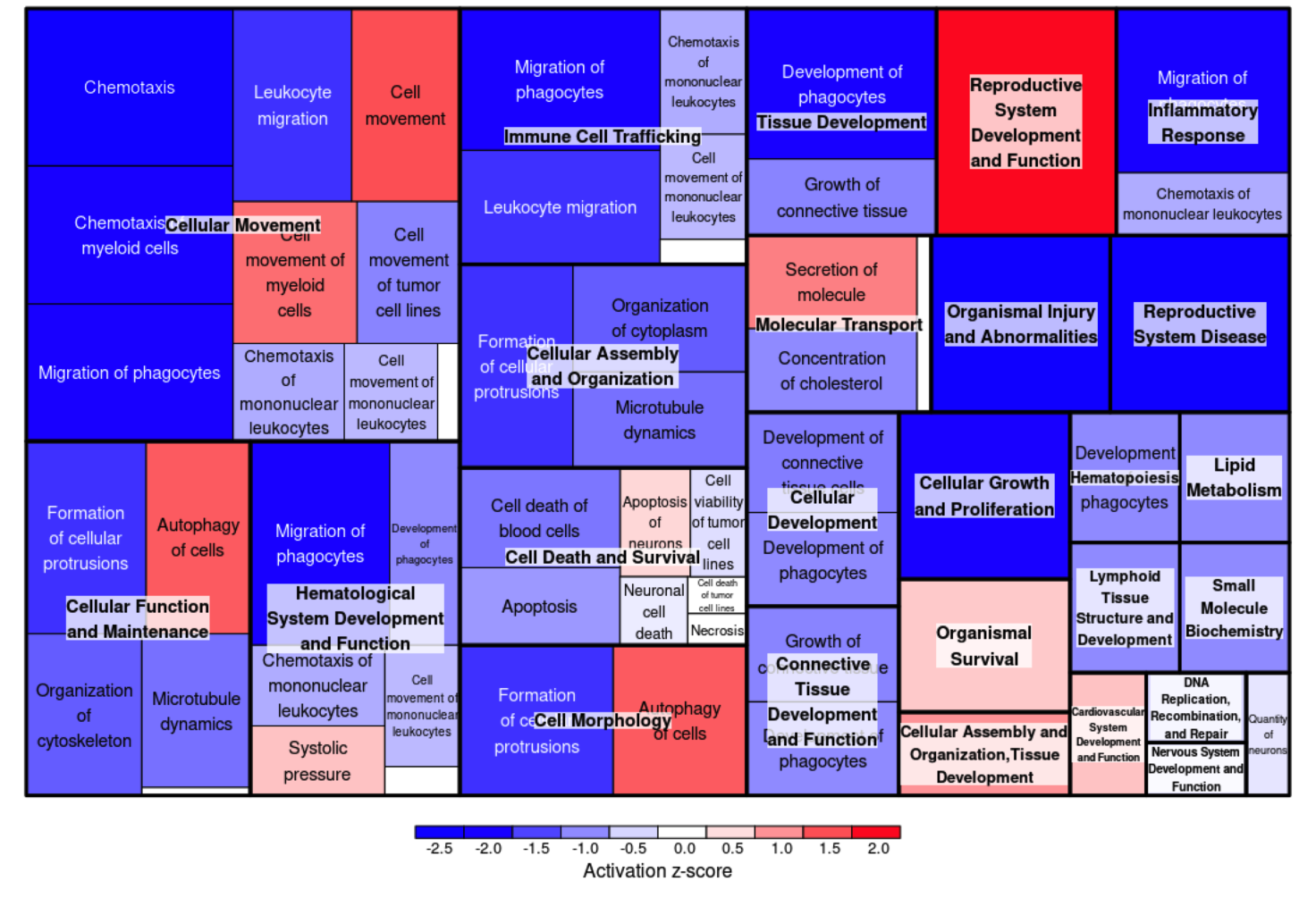

Supplement: Supplemental data [file Supp_FigS8.docx]
